# One-Year Comparative Outcomes of Scarf/Akin Osteotomy Versus Modified Lapidus Procedure in the Correction of Moderate to Severe Hallux Valgus

**DOI:** 10.7759/cureus.100680

**Published:** 2026-01-03

**Authors:** Amr Elfiky, Samir Hakeem, Mohammad Alkhreisat, Olive Kyaw, Chan Khin, Ahmed Elattar

**Affiliations:** 1 Department of Orthopaedic Surgery, University Hospitals Sussex NHS Foundation Trust, Brighton, GBR; 2 Department of Trauma and Orthopaedics, NHS Borders, Melrose, GBR; 3 Department of Special Surgery, Faculty of Medicine, Al-Balqa Applied University, Al Salt, JOR; 4 Department of Trauma and Orthopaedics, University Hospitals Sussex NHS Foundation Trust, Brighton, GBR; 5 Department of Trauma and Orthopaedics, Airedale NHS Foundation Trust, Keighley, GBR

**Keywords:** bunion, foot surgery, hallux valgus, lapidus procedure, outcomes, scarf osteotomy

## Abstract

Background: Hallux valgus is a common forefoot deformity. Both Scarf/Akin osteotomy and the modified Lapidus procedure (MLP) are established surgical options for moderate to severe cases, but few studies directly compare outcomes.

Methods: A retrospective cohort study of patients undergoing Scarf/Akin osteotomy or MLP between 2019 and 2020 was performed. Patient-reported outcomes (Manchester Oxford Foot Questionnaire (MOXFQ), Foot and Ankle Outcome Scores (FAOS), EQ-5D-5L) and radiographic measures (hallux valgus angle (HVA), intermetatarsal angle (IMA)) were compared at a minimum 12-month follow-up.

Results: Thirty-one patients were included (22 Scarf/Akin, nine MLP; mean age 58 years; 23 female patients). Patients in the Scarf group reported less difficulty with walking/standing (MOXFQ, p = 0.042) and higher activities of daily living (FAOS ADL, p = 0.049). EQ-5D demonstrated trends toward fewer mobility and self-care problems in Scarf patients, but differences were not statistically significant. Radiographically, MLP achieved greater correction of HVA (p = 0.011) and IMA (p < 0.001).

Conclusion: Both procedures significantly improved outcomes at one year. Scarf/Akin osteotomy was associated with better short-term functional recovery, while MLP achieved superior radiographic correction. Procedure choice may be individualized based on patient factors and deformity severity.

## Introduction

Hallux valgus (HV), or bunion, is one of the most common forefoot deformities, characterized by medial prominence at the first metatarsophalangeal (MTP) joint. HV affects up to one-third of adults, with prevalence increasing with age and female sex [1]. Pain is the primary symptom, often accompanied by erythema and inflammation from bursitis over the medial eminence [2]. Laxity of the supporting ligaments, particularly around the first and fifth metatarsal heads, can increase the intermetatarsal angle (IMA) beyond the normal 10°, accentuating valgus deviation of the great toe toward the second toe [3].

HV is multifactorial, with associations including genetic predisposition, foot morphology, footwear, and postural sway [4,5]. In particular, narrow or high-heeled footwear has been shown to exacerbate deformity and forefoot loading patterns [6]. Management options range from non-operative to surgical, depending on severity. Among surgical techniques, the Scarf osteotomy and the Lapidus procedure are widely employed.

The Lapidus procedure, originally popularized by Paul W. Lapidus, involves arthrodesis of the first tarsometatarsal (TMT) joint and is typically reserved for moderate to severe deformities, particularly with joint hypermobility, ligamentous laxity, or recurrence [7,8]. Modern modifications, such as the triplanar correction described by Shah et al., address both IMA and hallux valgus angle (HVA) [9].

The Scarf osteotomy offers stability and ease of fixation, traditionally used in younger patients with moderate deformity and good bone stock [10], though its use has been extended to more severe cases (HVA ≤ 40°, IMA ≤ 20°) [11].

Despite their frequent use, few studies directly compare these two techniques. Prior reports suggest longer recovery with Lapidus, higher recurrence after Scarf-Akin compared with Lapidus-Akin [12], and greater radiographic correction with modified Lapidus, though with similar patient-reported outcomes [13].

This study aims to compare clinical, radiographic, and patient-reported outcomes between the modified Lapidus procedure (MLP) and Scarf osteotomy in symptomatic HV, using three validated tools: the Manchester Oxford Foot Questionnaire (MOXFQ), EQ-5D (UK), and Foot and Ankle Outcome Scores (FAOS).

## Materials and methods

Study design

This is a retrospective cohort study with prospectively maintained data. All patients who underwent surgical correction of primary HV by one of five fellowship-trained foot and ankle orthopedic surgeons at our institution between January 2019 and December 2020 were reviewed.

Inclusion and exclusion criteria

Patients were eligible if they were 18 years of age or older at the time of surgery, underwent a primary MLP or Scarf/Akin osteotomy for HV, had an IMA greater than 11°, had both preoperative and at least one-year postoperative patient-reported outcome measures available, and had weightbearing anteroposterior (AP) and lateral radiographs at baseline and at least three months postoperatively. Exclusion criteria included refusal to provide consent, prior reconstructive surgery of the same foot, revision procedures, or concomitant major midfoot or hindfoot surgery such as hindfoot fusion or flatfoot reconstruction.

Surgical procedures

The MLP was defined as corrective arthrodesis of the first TMT joint without extension to the base of the second metatarsal [13]. The Scarf/Akin osteotomy was performed in standard fashion, with the Akin osteotomy added at the surgeon’s discretion for residual HV.

Outcomes and measurements

Radiographic outcomes included HVA and IMA, measured on standardized weight-bearing AP radiographs by independent reviewers. The radiographic parameters and measurement methods followed those described in standard orthopedic references [14].

Patient-reported outcomes were assessed using three validated instruments: the MOXFQ [15], EQ-5D (UK) [16], and the FAOS [17].

Follow-up questionnaires were administered at a minimum of six months, with final analysis performed at ≥12 months postoperatively.

Statistical analysis

Continuous variables were reported as mean ± standard deviation (SD). Between-group comparisons were made using independent-sample t-tests for normally distributed variables. A p-value <0.05 was considered statistically significant, and 95% confidence intervals (CI) were calculated. Statistical analysis was performed using SPSS, version 27 (IBM Corp., Armonk, NY).

## Results

A total of 31 patients were included in the study. The mean age was 58 years, with 23 female and eight male patients. Of these, 22 patients underwent Scarf/Akin osteotomy and nine underwent an MLP.

MOXFQ outcomes

Patients in the Scarf group reported significantly less difficulty with walking/standing compared with the MLP group (18.7 ± 18.3 vs 39.3 ± 36.0, p = 0.042). No significant differences were observed between groups for pain or social interaction domains (Table 1).

**Table 1:** MOXFQ outcomes at one-year follow-up MLP, modified Lapidus procedure; MOXFQ, Manchester Oxford Foot Questionnaire.

Domain	MLP Mean ± SD	Scarf Mean ± SD	p-Value
Walking/Standing	39.3 ± 36.0	18.7 ± 18.3	0.042
Pain	35.6 ± 32.2	23.6 ± 20.9	0.229
Social interaction	31.9 ± 32.4	16.5 ± 15.6	0.081

FAOS outcomes

Patients in the Scarf group demonstrated significantly higher scores in the activities of daily living (ADL) domain compared with the MLP group. No significant differences were observed in the other FAOS domains (Table 2). Overall, FAOS scores for pain, return to sport, and quality-of-life domains did not differ significantly between groups (p > 0.05).

**Table 2:** FAOS outcomes at one-year follow-up MLP, modified Lapidus procedure; ADL, activities of daily living; FAOS, Foot and Ankle Outcome Scores.

Domain	MLP Mean ± SD	Scarf Mean ± SD	p-Value
Symptoms	73.8 ± 30.6	81.6 ± 13.2	0.351
Pain	69.8 ± 35.9	82.1 ± 17.2	0.202
ADL	71.2 ± 33.7	90.7 ± 13.0	0.049
Sports and recreational activities	62.5 ± 47.9	45.0 ± 31.2	0.477
Quality of life	59.4 ± 39.5	71.4 ± 22.3	0.306

EQ-5D outcomes

**Figure 1:**
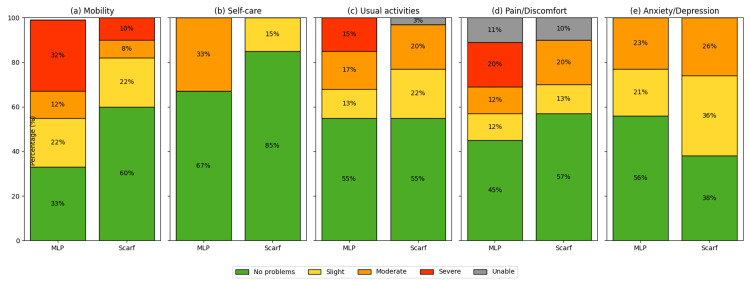
EQ-5D-5L outcomes by domain a) Mobility, (b) self-care, (c) usual activities, (d) pain/discomfort, and (e) anxiety/depression. Bars represent the proportion of patients reporting each level of severity in the modified Lapidus (MLP) and Scarf groups. Scarf patients more frequently reported “no problems” in mobility and self-care, whereas MLP patients reported a higher proportion of severe difficulties. Figure created by the authors based on EQ-5D-5L dimensions [16] (© EuroQol Research Foundation. EQ-5D™ is a registered trademark of the EuroQol Research Foundation). MLP, modified Lapidus procedure.

Analysis of EQ‑5D dimensions showed no significant differences between groups across mobility, ADL, and pain domains (p > 0.05). Descriptively, a greater proportion of patients in the Scarf group reported “no problems” in mobility and self‑care, whereas a higher proportion of MLP patients reported severe difficulties in these domains, although these differences did not reach statistical significance (Figure 1).

Radiographic outcomes

The MLP group demonstrated significantly greater correction of both the HVA and the IMA compared with the Scarf group (Table 3). The Lapidus group achieved significantly greater correction of both the HVA and the IMA compared with the Scarf group (HVA correction 28.1 ± 15.3° vs 17.3 ± 7.2°, p = 0.011; IMA correction (MLP value)° vs (Scarf value)°, p < 0.001).

**Table 3:** Radiographic outcomes at one-year follow-up MLP, modified Lapidus procedure; HVA, hallux valgus angle; IMA, intermetatarsal angle.

Variable	MLP Mean ± SD	Scarf Mean ± SD	p-Value
HVA correction (°)	28.1 ± 15.3	17.3 ± 7.2	0.011
IMA correction (°)	69.3 ± 25.4	72.1 ± 13.7	<0.001

## Discussion

This study compared outcomes of the MLP and Scarf/Akin osteotomy for the treatment of moderate to severe HV using three validated patient-reported outcome measures (MOXFQ, FAOS, and EQ-5D-5L), along with radiographic assessment. To our knowledge, this is the first study to simultaneously evaluate these two surgical techniques using this combination of clinical, radiographic, and health-related quality-of-life measures.

The MOXFQ is a dedicated instrument for HV, validated for use in clinical trials and surgical outcomes research [15,18]. Its psychometric properties and reproducibility have been demonstrated in multiple studies [19]. In our cohort, Scarf patients reported significantly less difficulty with walking and standing compared with MLP, while no differences were noted for pain or social domains. Similarly, FAOS results demonstrated higher ADL scores in the Scarf group, though other domains were comparable between groups. These findings suggest that patients undergoing Scarf osteotomy may experience superior functional mobility in the short term, despite broadly similar overall patient-reported outcomes.

When comparing our results to prior studies, similar trends emerge. Pinney et al. surveyed surgeons regarding preferred procedures for severe HV and found no consensus [20]. More recently, Reilly et al. reported no significant differences in patient-reported outcomes between MLP and Scarf osteotomy at one year, although the Lapidus procedure achieved greater radiographic correction [13]. Our findings align with these results, suggesting that both procedures achieve meaningful clinical improvements, but Lapidus offers superior angular correction while Scarf may be associated with earlier functional recovery. Similar long-term comparisons have demonstrated sustained correction with the Lapidus procedure and comparable satisfaction at 5-10 years [21].

EQ-5D analysis provided further insights. The EQ-5D instrument is widely used in orthopedic outcomes research and allows for conversion into quality-adjusted life year (QALY) estimates [22]. No statistically significant differences were found between groups; however, descriptively, patients undergoing Scarf more often reported “no problems” in mobility and self‑care, while MLP patients more frequently reported severe problems, in keeping with the MOXFQ and FAOS ADL findings. Nevertheless, the overall EQ-5D index and general health scores were comparable, suggesting that both procedures provide similar improvements in health-related quality of life.

Radiographically, the MLP achieved significantly greater correction of both the HVA and the IMA compared with the Scarf osteotomy. This is consistent with previous literature describing the Lapidus procedure as more powerful in addressing severe deformity and in controlling first-ray hypermobility [8,9,13]. Over time, various modifications, such as cross-screw fixation and triplanar correction, have improved union rates and stability [23]. Despite this, the greater correction did not translate into superior patient-reported outcomes at one year, raising the possibility that radiographic improvements may not directly correlate with functional outcomes in the short term. This supports previous findings that radiographic correction does not always correlate with patient-perceived functional improvement [24].

Limitations

Several limitations should be acknowledged. First, the sample size was relatively small (n = 31), with only nine patients in the MLP group, which limits statistical power and may explain some of the nonsignificant findings. Second, the retrospective study design is subject to potential selection bias and limits causal inference. Third, follow-up was limited to one year; recurrence rates and long-term functional outcomes may differ and were not captured in this analysis. Fourth, radiographic measurements were not performed by blinded independent assessors, which could introduce measurement bias. Finally, the use of multiple outcome instruments increases the robustness of findings but also raises the possibility of type I error due to multiple comparisons. Recurrence remains multifactorial, with preoperative deformity severity, sesamoid position, and first-ray hypermobility identified as key predictors.

Recommendations

Future research should focus on larger prospective, randomized controlled trials with long-term follow-up to better compare functional outcomes, recurrence, and durability of correction between these two procedures. Standardized radiographic assessment and cost-effectiveness analyses would further strengthen the evidence base. Additionally, stratification by deformity severity and patient characteristics (e.g., bone quality, ligamentous laxity, activity level) may help guide personalized surgical decision-making.

These conclusions should be viewed cautiously in light of the small and imbalanced sample and the retrospective design, and require confirmation in larger prospective studies.

## Conclusions

Both procedures provided significant improvement at one year, with better functional scores in the Scarf group and greater radiographic correction in the Lapidus group; these exploratory findings require validation in larger, adequately powered comparative studies.
